# Tissue inhibitor of metalloproteinase-1 (TIMP-1) as a prognostic biomarker in gastrointestinal cancer: a meta-analysis

**DOI:** 10.7717/peerj.10859

**Published:** 2021-02-16

**Authors:** Lili Qin, Yueqi Wang, Na Yang, Yangyu Zhang, Tianye Zhao, Yanhua Wu, Jing Jiang

**Affiliations:** Division of Clinical Research, First Hospital of Jilin University, Changchun, Jilin, China

**Keywords:** Tissue inhibitor of metalloproteinase 1, Immunohistochemistry, Enzyme linked immunosorbent assay, Gastric cancer, Colorectal cancer, Prognosis, Meta-analysis

## Abstract

**Background:**

Tissue inhibitor of metalloproteinase 1 (TIMP-1) has recently been shown to be dependent on or independent of Matrix metalloproteinases (MMPs) in its roles in tumorigenesis and progression. This appreciation has prompted various studies assessing the prognostic value of TIMP-1 in patients with gastrointestinal cancer, however, the conclusions were still inconsistent. The aim of this study was to assess the prognostic value of TIMP-1-immunohistochemistry (IHC) staining and pretreatment serum/plasma TIMP-1 level in gastrointestinal cancer survival as well as the association between TIMP-1 and clinicopathologic features.

**Methods:**

The meta-analysis was registered in the International Prospective Register of Systematic Reviews (PROSPERO; Registration NO. CRD42020185407) and followed the Preferred Reporting Items for Systematic Reviews and Meta-analyses (PRISMA) statement. A highly sensitive literature search was performed in electronic databases including PubMed, EMBASE and the Cochrane Library. Heterogeneity analysis was conducted using both chi-square-based Q statistics and the I^2^ test. The pooled hazard ratios (HRs) with 95% confidence intervals (CIs) were calculated to assess the prognostic value of TIMP-1 using the fixed-effects model. Odds ratios (ORs) with 95% CIs were calculated to evaluate the associations between TIMP-1 and clinicopathological characteristics. The meta-analysis was conducted using STATA 12.0 software.

**Results:**

A total of 3,958 patients from twenty-two studies were included in the meta-analysis. Elevated TIMP-1 levels were significantly associated with poor survival in gastrointestinal cancer (TIMP-1-IHC staining: HR = 2.04, 95% CI [1.59–2.61], *I*^2^ = 35.7%, *P*_Q_ = 0.156; pretreatment serum/plasma TIMP-1 levels: HR = 2.02, 95% CI [1.80–2.28], *I*^2^ = 0%, *P*_Q_ = 0.630). Moreover, clinicopathological parameter data analysis showed that elevated TIMP-1 levels were significantly associated with lymph node metastasis (N1/N2/N3 vs N0: OR = 2.92, 95% CI [1.95–4.38]) and higher TNM stages (III/IV vs I/II: OR = 2.73, 95% CI [1.23–6.04]).

**Conclusion:**

Both TIMP-1-positive IHC staining and high serum/plasma TIMP-1 levels are poor prognostic factors for the survival of gastrointestinal cancer. In addition, TIMP-1 overexpression was correlated with more advanced clinicopathological features.

## Introduction

Gastric cancer (GC) and colorectal cancer (CRC) are the fifth and third most common types of cancers worldwide (Bray et al., 2018). Despite advances in the diagnosis and treatment of gastrointestinal cancer in recent decades, the prognosis for gastrointestinal cancer patients is still poor. Studies have shown that invasion and metastasis are the most important prognostic factors in gastrointestinal cancer (Jiang et al., 2015; Yasui et al., 2005), which highlights the importance of identifying invasion and metastasis-associated prognostic markers to guide clinical practice and explain the variability of survival.

The degradation of the extracellular matrix (ECM) is a critical part of tumour cell invasion and metastasis, and both matrix metalloproteinases (MMPs) and tissue inhibitors of metalloproteinases (TIMPs) have been confirmed to be involved in this process. Among all TIMPs members, TIMP-1 has been recognized as the most promising marker in tumorigenesis and progression since its unique two-domain structure harboring metalloproteinase-inhibitory and cytokine-like signaling activities (Grünwald, Schoeps & Krüger, 2019). Moreover, TIMP-1 is the only N-linked glycoprotein with glycosylation sites at N30 and N78 which can exert potent biological functions (Kim et al., 2012). Furthermore, relative to other TIMPs, widespread TIMP-1 increase can be observed in solid cancers as tumours progress (Jackson et al., 2017). With respect to its MMP-dependent functions, TIMP-1 can play a negative role in tumour cell adhesion and inhibit the degradation of ECM and basement membranes facilitated by MMPs (Bourboulia & Stetler-Stevenson, 2010). Regarding its MMP-independent functions, TIMP-1 can bind with cytokines, adhesion molecules, cell surface proteins and induce survival signals, simultaneously affecting tumour architecture and progression. Several studies have suggested that TIMP-1 can stimulate cell proliferation (Bigelow et al., 2009; Hayakawa et al., 1992), inhibit apoptosis (Liu et al., 2003; Liu et al., 2005), induce angiogenesis (Kessenbrock, Plaks & Werb, 2010), accelerate tumour invasion and metastasis (D’Angelo et al., 2014), and cause adverse cancer hallmarks via crucial signals, such as the regulation of NOTCH and WNT (Jackson et al., 2017) and participation in transforming growth factor-*β* (TGF*β*)-regulated crosstalk (Park et al., 2015). Based on its complex and controversial functions, the role of TIMP-1 in tumour progression is still debated.

A comprehensive literature search showed that TIMP-1 expression in breast cancer, GC and CRC was strongly upregulated compared to that in other cancers (Jackson et al., 2017), which indicated that TIMP-1 might play a more important role in gastrointestinal cancer. Although a recent meta-analysis of original reports demonstrated the poor prognostic value of TIMP-1-positive expression in solid cancers (Lee, Choi & Kim, 2011; Liu et al., 2019), the article included only three original studies on gastrointestinal cancer. In addition, all studies included in this meta-analysis used IHC but not ELISA to assess TIMP-1 expression in cancer patients. Moreover, it did not explore the associations between TIMP-1 and clinicopathological parameters. In addition, based on the potential inhibitory effect on chemotherapy-induced apoptosis, TIMP-1 serves as an effective biomarker to predict the response to chemotherapy (chemo) in CRC and has attracted tremendous attention, but no consistent conclusion has yet been reached (Frederiksen et al., 2011; Spindler et al., 2015; Unsal et al., 2008). Therefore, it is important to conduct a meta-analysis restricted to gastrointestinal cancer to quantitatively appraise the prognostic value of TIMP-1-IHC staining and pretreatment serum/plasma TIMP-1 levels and reach a conclusion about the association between TIMP-1 and the survival of gastrointestinal cancer patients with different clinical characteristics.

## Materials & Methods

### Search strategy

The meta-analysis was registered in the International Prospective Register of Systematic Reviews (https://www.crd.york.ac.uk/prospero/display_record.php? ID=CRD42020185407) and followed the Preferred Reporting Items for Systematic Reviews and Meta-analyses (PRISMA) statement (Moher et al., 2010). PubMed, EMBASE and the Cochrane Library were systematically searched for relevant articles published before March 15, 2020. The key words used were as follows: “Tissue Inhibitor of Metalloproteinase 1 OR TIMP-1” AND “Stomach Neoplasms OR gastric cancer OR gastric carcinoma OR stomach cancer” OR “Colorectal Neoplasms OR colorectal cancer OR colon cancer OR rectal cancer” AND “prognosis OR survival OR outcome OR prognostic” AND “serum OR plasma OR enzyme-linked immunosorbent assay OR ELISA OR Immunohistochemistry OR IHC”. In addition, we searched reference lists from identified primary studies and review articles to identify additional eligible studies missed by the electronic search strategies. All enrolled studies were restricted to publication in English.

### Study selection

Two reviewers (Lili Qin, Yueqi Wang) performed the selection process independently, and any discrepancies were resolved upon discussion until a consensus was reached or following the third reviewer’s decision (Yanhua Wu). Among the records in the primary search, overlapping articles were excluded by browsing the authors’ names and affiliations. Irrelevant studies were excluded by screening the titles and abstracts. Studies included in the meta-analysis met the following criteria: (1) all patients were diagnosed with GC or CRC; (2) studies assessed the association between TIMP-1 and the overall survival (OS) of patients; (3) TIMP-1 was evaluated using an enzyme-linked immunosorbent assay (ELISA) or immunohistochemical (IHC) method; (4) hazard ratios (HRs) and 95% confidence intervals (CIs) could be obtained from the article. The exclusion criteria were as follows: (1) review articles or case reports; (2) patients who received preoperative anticancer treatment; (3) repeated articles published with the same cohort of patients.

### Data extraction

Eligible data were extracted by two researchers independently, including the first author’s surname, publication year, geographical location, sample size, mean/median age, median follow-up period, positive ratio, sex ratio, tumour type, tumour stage, method of TIMP-1 measurement, detection method, five-year survival rate, cut-off value used for assessing TIMP-1 positivity, and HRs and 95% CIs for OS. For articles lacking survival data, HRs and 95% CIs were extracted from survival curves using Engauge Digitizer version 4.1. Any differences in the data extraction were resolved by the two researchers.

### Quality assessment

The quality was evaluated by two observers using the Newcastle-Ottawa Scale (NOS) criteria (Stang, 2010). The NOS criteria included three aspects: (1) subject selection: 0–4; (2) comparability of subjects: 0–2; and (3) clinical outcome: 0–3. NOS scores ranged from 0 (the lowest) to 9 (the highest), and a score ≥6 indicates high quality.

### Statistical analysis

The pooled HRs with 95% CIs were calculated to assess the prognostic value of TIMP-1 in gastrointestinal cancer survival, and the odds ratio (OR) and corresponding 95% CIs were used to report the aggregated association strength between TIMP-1 and clinicopathological characteristics.

Heterogeneity among studies was assessed using Cochran’s Q statistic and I^2^ tests, and *P* < 0.05 in the Q-test or I^2^ >50% was considered to be statistically heterogeneous. If there was significant heterogeneity among the studies, the random-effects model was used to conduct the analysis; otherwise, the fixed-effects model was used. Sensitivity analysis was performed by the successive omission of each study to assess the integrity of the summary results. Publication bias was examined by funnel plots and Egger’s test. All of the analyses were two-sided, and *P* < 0.05 was considered to be statistically significant. STATA statistical software (Version 12.0; Stata Corporation, College Station, TX) was used to conduct the analyses.

## Results

### Literature information and characteristics

**Figure 1:**
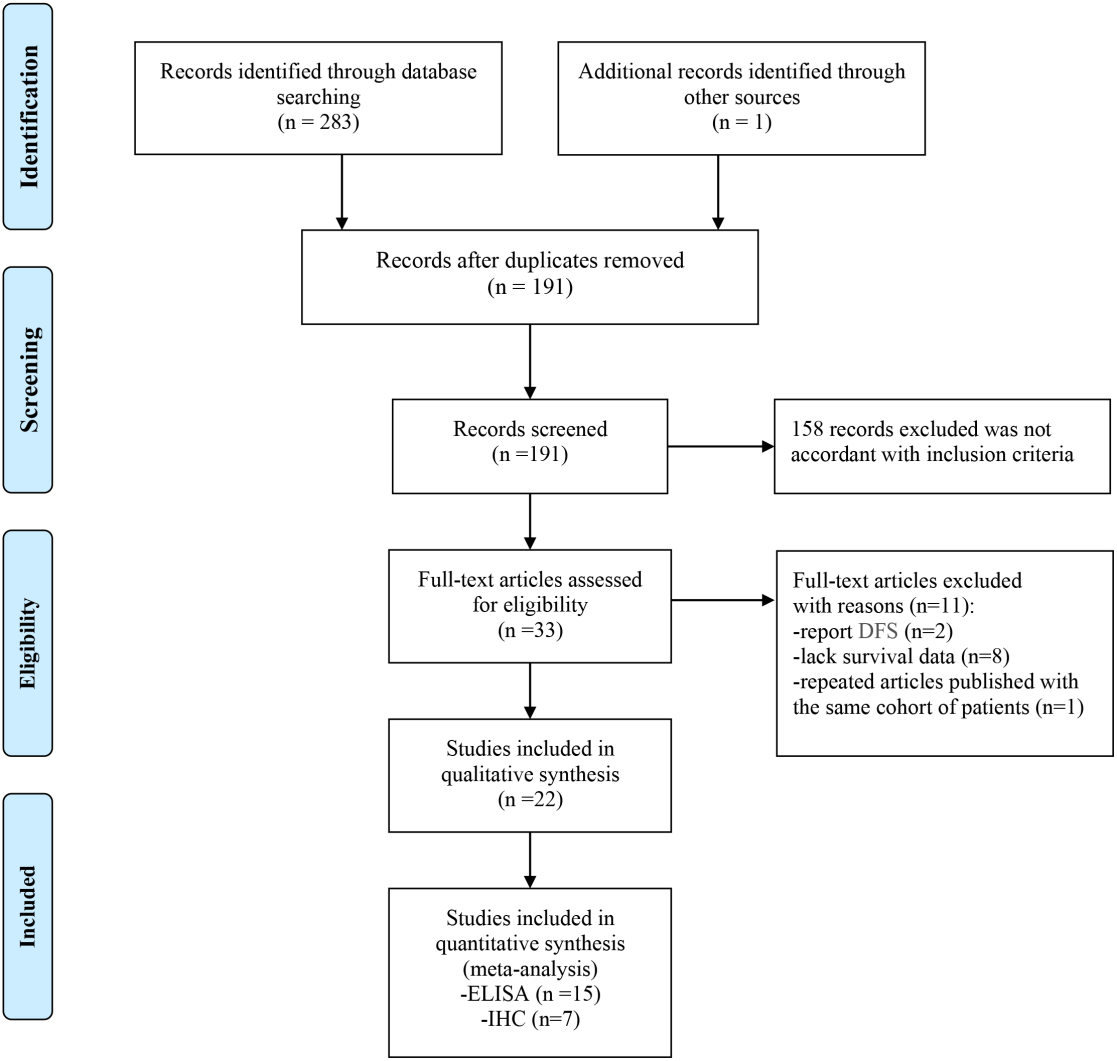
Flow chart of the enrolled studies.

The flow chart of the meta-analysis is shown in Fig. 1. A total of 283 studies were identified using the search strategy, and 1 study was identified through other sources. Ninety-three duplicated studies were excluded, and 158 irrelevant studies were excluded after screening the titles and abstracts. Among 33 studies that were further evaluated, 11 studies were also excluded due to a lack of survival data (*n* = 8) or the evaluation of disease-free survival (*n* = 2) or publication with the same cohort of patients (*n* = 1). Finally, 22 studies with a total of 3958 patients were enrolled in this study.

The main characteristics of the studies evaluating the prognostic value of TIMP-1-IHC staining and pretreatment serum/plasma TIMP-1 levels are shown in Tables 1 and 2, respectively. The publication year ranged from 1999 to 2019, and the sample size ranged from 54 to 588. Seven studies utilized the IHC method (Jensen et al., 2010; Joo et al., 1999; Li et al., 2016; Mroczko et al., 2009; Roca et al., 2006; Song et al., 2016; Unsal et al., 2008), and others used ELISA (Aldulaymi et al., 2010; Birgisson et al., 2010; Böckelman et al., 2018; Byström et al., 2012; Frederiksen et al., 2011; Giaginis et al., 2009; Holten-Andersen et al., 2004; Holten-Andersen et al., 2000; Laitinen et al., 2018; Oblak et al., 2013; Spindler et al., 2015; Vočka et al., 2019; Wang et al., 2006; Yoshikawa et al., 2009; Yukawa et al., 2007). TIMP-1-positive IHC staining was defined as a tumour cell positive percentage ≥25% (*n* = 1), weak or moderate staining intensity (*n* = 4) or immune risk score (IRS) ≥2 (*n* = 2). The cut-off value of the pretreatment serum/plasma TIMP-1 level ranged from 102 to 600 ng/ml. Among studies evaluating the prognostic value of TIMP-1 IHC staining, 4 studies estimated univariate HRs and 3 studies obtained multivariate HRs; 3 studies originated from Asia and 4 from Europe. Among studies evaluating the prognostic value of pretreatment serum/plasma TIMP-1 levels, 5 studies estimated univariate HRs and 10 studies obtained multivariate HRs; 3 studies originated from Asia and 12 from Europe. In addition, of all eligible studies, 6 studies included metastatic CRC patients (Aldulaymi et al., 2010; Byström et al., 2012; Frederiksen et al., 2011; Spindler et al., 2015; Unsal et al., 2008; Vočka et al., 2019), and 1 study recruited nonmetastatic patients (Oblak et al., 2013). 8 studies were conducted among CRC patients with chemotherapy and 3 studies among nonchemotherapy patients. For the NOS criteria, the scores of all included studies were ≥6 and showed high quality.

**Table 1:** Studies evaluating the prognostic value of TIMP-1-IHC staining.

**Author (Year)**	**Country**	**Sample**	**Tumor style**	**Follow-up (months)**	**Median age**	**Male/ Female**	**Chemotherapy**^ g^	**Metastasis**^h^	**Cut-off (IHC)**	**HR(95% CI)**	**NOS score**
Joo et al. (1999)	Korea	54	CRC	NS	59.8 ±12.0^b^	32/22	No^e^	No^a^	intensity>0	1.83(0.53–6.52)^c^	6
Roca et al. (2006)	Italy	84	CRC	60	NS	47/37	5-FU and Leukovorin	No^a^	≥25%	2.57(1.01–6.54)^c^	8
Unsal et al. (2008)	Turkey	60	CRC	29.45^a^	NS	37/23	5-FU and leucovorin	Yes	IRS ≥2	0.64(0.15–2.71)^c^	6
Mroczko et al. (2009)	Poland	54	GC	48	67.5	41/13	NS	No^a^	intensity>0	4.58(1.86–10.83)^c^	6
Jensen et al. (2010)	Denmark	340	CRC	72	NS	170/170	5-FU and isovorin	No^a^	Intensity 2-3	1.60(1.10–2.20)^d^	9
Li et al. (2016)	China	329	CRC	58	62	122/207	No^e^	No^a^	intensity>0	2.64 (1.87–5.82)^d^	6
Song et al. (2016)	China	94	CRC	NS	67	47/47	No	No^a^	IRS ≥2	2.91(1.25–6.74)^d^	9

**Notes.**

a Median follow-up.

b Mean age.

c Univariate analysis.

d Multivariate analysis.

e Not all patients received chemotherapy.

f Not all patients with metastatic cancer.

g All recruited patients stratified by chemotherapy.

h All recruited patients stratified by metastasis.

NS Data were not shown

**Table 2:** Studies evaluating the prognostic value of pre-treatment serum/plasma TIMP-1 level (ELISA).

**Author (Year)**	**Country**	**Sample**	**Material**	**Follow-up (months)**	**Median sge**	**Male/ Female**	**Chemo**^g^	**Cut-off (ELISA)**	**HR (95% CI)**	**NOS score**
Holten-Andersen et al. (2000)	Denmark	588	Plasma	81.6	69^b^	352/236	No	NS	2.50(1.70–3.70)^d^	7
Holten-Andersen et al. (2004)	Sweden	352	Plasma	43.0	68^b^	226/126	No^e^	196 ng/ml	2.20(1.20–4.10)^d^	7
Wang et al. (2006)	Taiwan	170	Serum	NS	65.1	112/58	NS	239.1 ng/ml	1.84(1.13–2.98)^c^	6
Yukawa et al. (2007)	Japan	87	Plasma	70.0	NS	54/33	NS	170 ng/ml	2.13(0.93–4.90)^d^	7
Yoshikawa et al. (2009)	Japan	149	Plasma	63.9	NS	103/46	NS	112.5 ng/ml	2.29(1.28–4.09)^d^	8
Giaginis et al. (2009)	Greece	97	Serum	20.0	66.47^ b^	54/43	No	260.23 ng/mL	2.44(1.20–4.98)^d^	8
Birgisson et al. (2010)	Sweden	322	Plasma	78.0	73^b^	163/159	No^e^	NS	1.80(1.30–2.40)^d^	7
Aldulaymi et al. (2010)	Nordic	88	Plasma	NS	NS	NS	FOLFIRI	NS	3.80(2.40–6.00)^c^	6
Frederiksen et al. (2011)	Nordic	120	Plasma	34.0	65	69/51	FOLFOX	208 ng/ml	1.80(1.17–2.78)^c^	7
Byström et al. (2012)	Nordic	106	Serum Plasma	NS	60	67/39	FOLFIRILv5FU2-CPT11	111 ng/mL	2.10(1.11–3.94)^d^	7
Oblak et al. (2013)	Slovenia	92	Plasma	68.0	73	63/29	5-FU leukovorin	170 ng/ml	2.15(1.01–4.56)^d^	7
Spindler et al. (2015)	Denmark	107	Plasma	36.0	62	58/49	irinotecan cetuximab	NS	1.83(1.29–2.59)^d^	7
Laitinen et al. (2018)	Finland	233	Serum	NS	67.4	152/161	No^e^	170 ng/ml	1.85(1.26–2.72)^d^	6
Böckelman et al. (2018)	Finland	335	Serum	76.8^a^	67.2^b^	174/161	NS	151 ng/ml	1.80(1.23–2.64)^c^	7
Vočka et al. (2019)	Czech Republic	97	Serum	NS	64.4	60/37	NS	600 ng/ml	1.65(1.07–2.54)^c^	6

**Notes.**

a Median follow-up.

b Mean age.

c Univariate analysis.

d Multivariate analysis.

e Not all patients received chemotherapy.

f Not all patients with metastatic cancer.

g All recruited patients stratified by chemotherapy.

h All recruited patients stratified by metastasis.

NS Data were not shown

### Association between TIMP-1 and OS

**Figure 2:**
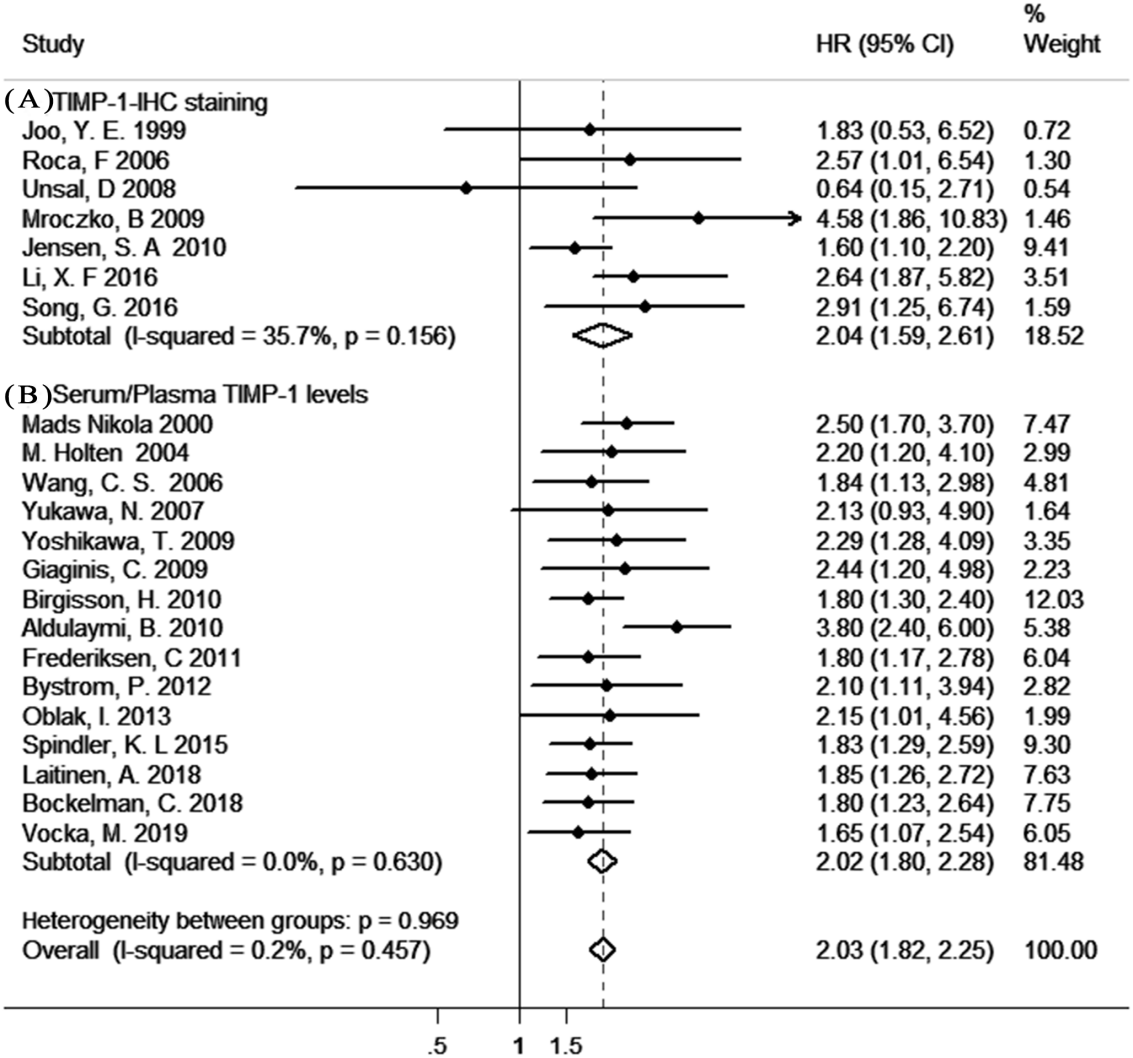
The results of the meta-analysis with studies evaluating TIMP-1 for OS in gastrointestinal cancer. (A) with the IHC method; (B) with the ELISA method.

As shown in Fig. 2, our results indicated a significant poor prognostic effect of TIMP-1 in gastrointestinal cancer survival with insignificant heterogeneity (TIMP-1-IHC staining: HR=2.04, 95% CI [1.59–2.61], *I*^2^ = 35.7%, *P*_Q_ = 0.156; pretreatment serum/plasma TIMP-1 levels: HR = 2.02, 95% CI [1.80–2.28], I^2^=0%, *P*_Q_ = 0.630). In addition, there was no significant difference in the pooled HR in the different TIMP-1-analytic method (IHC and ELISA) groups or HR analysis (univariate analysis and multivariate analysis) groups (Fig. S1).

Moreover, sensitivity analysis indicated no dominancy for any study (Figs. S2 –S3). Furthermore, there was no apparent asymmetry in the funnel plot (Fig. S4–S5); in support of this, Egger’s test showed no significant publication bias for evaluable studies (TIMP-1-IHC staining: Egger’s test *P* = 0.586; serum/plasma TIMP-1 levels: Egger’s test *P* = 0.258).

### Associations between TIMP-1 and clinicopathological parameters

The associations between TIMP-1 and clinicopathological characteristics were also evaluated (Table 3). The results showed that elevated TIMP-1 levels were significantly associated with lymph node metastasis (N1/N2/N3 vs N0, OR = 2.92, 95% CI [1.95–4.38]) and higher TNM stages (III/IV vs I/II, OR = 2.73, 95% CI [1.23–6.04]). No associations were found between high TIMP-1 levels and other clinicopathological characteristics, including age, gender, T stage, M stage, histological grade and vascular invasion.

**Table 3:** Meta-analysis of TIMP-1 overexpression association with clinicopathological parameters.

Parameter^a^	Number	OR (95% CI)	Z	*P*_Z_	*P*_Q_	I^2^
Age	473	1.20 (0.79–1.81)	0.86	0.389	0.780	0%^*^
Gender	1038	0.83 (0.64–1.07)	1.42	0.156	0.103	45.4%^*^
T stage	1015	1.60 (0.75–3.40)	1.21	0.227	0.001	74.7%
N stage	498	2.92 (1.95–4.38)	5.21	<0.001	0.750	0%^*^
M stage	330	2.99(0.63–14.25)	1.37	0.169	0.020	74.5%
TNM stage	262	2.73 (1.23–6.04)	2.48	0.013	0.099	52.2%
Histological grade	1098	1.56 (0.91–2.66)	1.62	0.105	0.004	69%
Vascular invasion	663	1.09 (0.78–1.52)	0.49	0.622	0.150	47.3%^*^

**Notes.**

OR pooled odds ratio CI confidence interval Z test value for fixed/random effect model *P*_*Z*_ statistical *P* value for Z test *P*_*Q*_ statistical *P* value for heterogeneity Q test I^2^ quantitative metric I^2^ test

a Male vs Female, T3/T4 vs T1/T2, N1/N2/N3 vs N0, M1vs M0, III/IV vs I/II, High histological grade vs Low histological grade, Positive vascular invasion vs Negative vascular invasion.

* Fixed-effects model.

### Subgroup analysis

To determine the prognostic value of TIMP-1 in gastrointestinal cancer survival in subgroups restricted to source country, metastasis and chemotherapy, we performed subgroup analyses (Table 4). Although none of the subgroups had significantly different pooled HRs compared to the overall groups or counterpart subgroups, stratified analyses restricted to Asia, Europe and metastatic patients indicated the poor prognostic value of high TIMP-1 levels in gastrointestinal cancer survival. In addition, regarding the role of pretreatment serum/plasma TIMP-1 levels in response to chemotherapy in CRC patients, high serum/plasma TIMP-1 levels tended to have higher numerical values of the pooled HR for CRC survival in the nonchemotherapy group, however, this difference was not statistically significant between the groups.

## Discussion

To date, multiple prognostic biomarkers of gastrointestinal cancer have been identified in recent researches so as to complement clinicopathological factors for individual therapy and the improvement of survival outcomes, such as, Microsatellite instability (MSI), human epidermal growth factor (HER), tumor suppressor gene (TP53, DCC, p27, RUNX3), carcinoembryonic antigen (CEA), pepsinogen C, invasion and metastasis-associated factor (uPA, E-cadherin, MMPs and TIMPs), apoptosis-associated factor (Bcl-2, Caspase 3, NF-KB) (Sawada et al., 2015; Yasui et al., 2005; Yiu & Yiu, 2016). Regrettably, there was no unified conclusion on their clinical utility due to methodological heterogeneity. Upon a cross-validation for previous gastrointestinal cancer survival biomarker candidates based on transcriptomic data, TIMP-1 was confirmed as one of the robustest candidate genes (Szasz et al., 2016). However, without confirmation at the protein level, the prognostic value is still limited. Our goal was to perform a robust meta-analysis enabling the swift evaluation.

**Table 4:** Subgroup meta-analysis results for TIMP-1 impact on gastrointestinal cancer survival.

Subgroups	Number	HR (95% CI)	Z	*P*_Z_	*P*_Q_	I^2^
All studies	3958	2.03 (1.82–2.25)	13.02	<0.001	0.457	0.2%^*^
Asia group	883	2.22 (1.70–2.91)	5.83	<0.001	0.920	0%^*^
Europe group	3075	1.99 (1.77–2.24)	11.67	<0.001	0.212	21.2%^*^
Metastasis^a^	578	2.01 (1.47–2.74)	4.40	<0.001	0.048	55.2%
Chemotherapy^b^	997	1.99 (1.67–2.38)	7.71	<0.001	0.090	43.2%^*^
No-chemo^c^	779	2.54 (1.85–3.49)	5.78	<0.001	0.942	0%^*^

**Notes.**

HR pooled hazard ratio CI confidence interval Z test value for fixed/random effect model *P*_*Z*_ statistical *P* value for Z test *P*_*Q*_ statistical *P* value for heterogeneity Q test I^2^ quantitative metric I^2^ test

* Fixed-effects model.

a CRC patients with metastasis.

b CRC patients with chemotherapy.

c CRC patients without chemotherapy.

To our knowledge, this is the first meta-analysis restricted to gastrointestinal cancer that investigated the prognostic value of TIMP-1 IHC staining and pretreatment serum/plasma TIMP-1 levels and explored the associations between TIMP-1 and clinicopathological characteristics. In the present study, a fixed-effects model was used to report the pooled HR due to the weak heterogeneity among the studies, and sensitivity analysis showed that the pooled HR was not affected by any individual study. Moreover, no publication bias was detected according to Egger’s test, which further strengthened the meta-analysis and indicated that our results are trustworthy.

Our research found that both TIMP-1-positive IHC staining and high pretreatment serum/plasma TIMP-1 levels were significantly associated with poor survival in gastrointestinal cancer. Although TIMP-1 has been expected to inhibit tumorigenesis, progression and metastasis by blocking the matrix-degrading properties of endopeptidases (Bourboulia & Stetler-Stevenson, 2010), its overexpression in tumour recently showed protease-independent roles in proliferation, anti-apoptosis, pro-angiogenesis, tumour invasion, metastasis and immune response-regulatory activities by interacting with cytokines, adhesion molecules, surface proteins and inducing critical survival signals (Jackson et al., 2017). More possible mechanisms of TIMP-1 in gastrointestinal cancer are related to MMP-independent functions. First, TIMP-1 can promote cell proliferation and inhibit apoptosis. It could bind to tetraspanin CD63 to drive cancer-associated fibroblast (CAF) accumulation, resulting in tumour growth (Gong et al., 2013; Jung et al., 2006). Meanwhile, the interaction of TIMP-1 and CD63 may enhance specific phosphorylation of both Akt and Bad (Bcl-2/Bcl-X (L)-antagonist, causing cell death) via focal adhesion kinase (FAK)/phosphoinositide 3-kinase (PI3K)-dependent survival signals, leading to the increased expression of the antiapoptotic protein Bcl-XL and inhibiting the caspase cascade (Lee et al., 2003; Liu et al., 2003; Sørensen et al., 2007). Second, TIMP-1 was reported to promote angiogenesis via activating vascular endothelial growth factor (VEGF) (Yoshiji et al., 1998). Notably, TIMP-1 is of crucial significance in cancer invasion and metastasis. modulation can be attributed to several events: Firstly, TIMP-1 targets cell adhesion molecules for disrupting cell–cell and cell–matrix adhesions, eg. TIMP-1 induces TWIST1 to downregulate E-cadherin, resulting in epithelial to mesenchymal transition (EMT) (D’Angelo et al., 2014). Secondly, TIMP-1 can help to create a metastatic niche by microRNA-210 (miR-210) regulation and blocking MET receptor shedding by ADAM10 (Cui et al., 2015; Schelter et al., 2011). Thirdly, TIMP-1 can function as a ligand itself and blind to tetraspanin CD63, and therefore activate FAK-PI3K/AKT and mitogen-activated protein kinase (MAPK) pathway (Song et al., 2016). Fourthly, the aberrant glycosylation of TIMP-1 contributes to high invasive potential of cancer cells in the tumor microenvironment (Kim et al., 2012). Other mechanisms include promotion of neovascularization and recruitment of tumour-associated immune cell via binding to cell–surface proteins or cytokines (Kobuch et al., 2015); (Tüting & De Visser, 2016). In support of these findings, our results showed that elevated TIMP-1 levels were associated with more advanced N stages and TNM stages, which suggested a greater possibility of metastasis. Furthermore, it has been extensively shown that TIMP-1 could cause adverse cancer hallmarks via other crucial signals, such as the mediation of receptor tyrosine kinases (RTKs) and proliferative signals (Miller et al., 2016), the regulation of NOTCH and WNT (Jackson et al., 2017), and participation in transforming growth factor-*β* (TGF- β)-regulated crosstalk (Park et al., 2015). Based on the abovementioned findings, it is plausible that the elevated expression of TIMP-1 is significantly associated with the poor survival of gastrointestinal cancer patients.

Currently, IHC and ELISA are recognized as the primary analytic methods for assessing TIMP-1 in gastrointestinal cancer. Although TIMP-1 protein levels are easily measured using ELISA, there is a substantial risk of false elevated levels due to the freezing and thawing of blood (Holten-Andersen et al., 1999); in contrast, immunohistochemical staining on histopathologic slides is intuitive and fast but invasive. To our knowledge, no study has investigated the consistency of the prognostic value of TIMP-1 for gastrointestinal cancer survival with the use of different analytic methods (IHC and ELISA). Our meta-analysis showed no significant difference in the TIMP-1 prognostic value between the two methods, which can be explained by the fact that the increases of TIMP-1 in blood may be a result of secretion from the cancer cells themselves (Holten-Andersen et al., 2002; Sørensen et al., 2007; Stephens et al., 1998). Accordingly, for non-operated patients or postoperative follow-up patients, using ELISA to evaluate TIMP-1 levels may be more convenient and quicker when assessing prognosis and conducting long-term monitoring. Additionally, the results showed no significant difference in the pooled HR between the univariate analysis and multivariate analysis groups, which indicated that the difference in the HR analysis method was not a significant source of heterogeneity.

Based on the finding that TIMP-1 can induce chemotherapy resistance in vivo by inhibiting apoptosis (Sørensen et al., 2007; Spindler et al., 2015), we analyzed the association between serum/plasma TIMP-1 levels and OS stratified by chemotherapy status. The results showed the poor prognostic value of high serum/plasma TIMP-1 levels in CRC survival in the chemotherapy group as well as in the non-chemotherapy group. Considering the diversity of the available chemotherapy regimens in the clinic, to assess the role of serum/plasma TIMP-1 levels in response to different chemotherapy regimens and overlapping combination therapies, further validation in randomized controlled trials stratified by types of chemotherapy will be essential in this setting. On the other hand, stratified analyses restricted to Asia, Europe and metastatic patients indicated the poor prognostic value of high TIMP-1 levels in gastrointestinal cancer survival, which implied that TIMP-1 may serve as a robust indicator for the prognosis of gastrointestinal cancer patients with different clinical stages or races.

Although we systematically performed subgroup analyses, there were still several limitations. First, we enrolled studies restricted to publication in English, which can overestimate the prognostic significance of TIMP-1 because positive studies tend to be published in English in contrast to negative studies (Earleywine, 1993; Egger et al., 1997). Second, although accurate and comprehensive literature searches were performed, the sample size in our study was still limited because most of included studies used small sample size. Third, there is currently still no general agreement upon a set of well-tested and validated antibodies, ELISA kits, evaluation criteria and protocols, which may influence the positive rate of TIMP-1. In addition, the difference in follow-up duration and endpoints in the included studies may also result in potential bias. Moreover, in the present study, the HR was extrapolated from survival curves for 3 studies, and 2 studies estimated the 95% CI using HR and *P* values in univariate analysis. Although we tried to increase the accuracy using a graphical curve reader software to read curves and choose appropriate time intervals, bias from data extraction still needs to be considered. Considering existing limitations, further attention should be paid to multicenter, larger scale and scientifically designed studies.

## Conclusions

In general, this meta-analysis of statistically homogenous data from 3958 patients investigated whether both TIMP-1-positive IHC staining and high serum/plasma TIMP-1 levels are poor prognostic factors for the survival of gastrointestinal cancer. Moreover, TIMP-1 overexpression was correlated with more advanced clinicopathological features. Therefore, TIMP-1 alone or an appropriate combination of TIMP-1 and other biomarkers would have great promise in the clinic.

##  Supplemental Information

10.7717/peerj.10859/supp-1 Supplemental Information 1 The HR with 95% CIs assessing the prognostic value of TIMP-1 in 22 enrolled studies Hr = hazard ratio; ll = lower confidence limit; ul = upper confidence limit.

10.7717/peerj.10859/supp-2 Supplemental Information 2 Clinicopathological characteristics of patients according to the TIMP-1 expression in 22 enrolled studies tevent and cevent: the number of patients with male, T3/T4, N1/N2/N3, M1, High histological grade, Positive vascular invasion in TIMP-1+ group and TIMP-1- group respectively; tnoevent and cnoevent: the number of patients with female, T1/T2, N0, M0, Low histological grade, Negative vascular invasion in TIMP-1+ group and TIMP-1- group respectively.

10.7717/peerj.10859/supp-3 Supplemental Information 3 Rationale and Contribution.

10.7717/peerj.10859/supp-4 Supplemental Information 4 PRISMA checklist.

10.7717/peerj.10859/supp-5 Supplemental Information 5 The results of the meta-analysis with studies evaluating TIMP-1 for OS in gastrointestinal cancer (A): univariate analysis; (B): multivariate analysis.

10.7717/peerj.10859/supp-6 Supplemental Information 6 Sensitivity analysis for studies evaluating TIMP-1-IHC staining for OS in gastrointestinal cancer

10.7717/peerj.10859/supp-7 Supplemental Information 7 Sensitivity analysis for studies evaluating pretreatment serum/plasma TIMP-1 levels for OS in gastrointestinal cancer

10.7717/peerj.10859/supp-8 Supplemental Information 8 Egger’s funnel plot of studies evaluating TIMP-1-IHC staining for OS in gastrointestinal cancer

10.7717/peerj.10859/supp-9 Supplemental Information 9 Egger’s funnel plot of studies evaluating pretreatment serum/plasma TIMP-1 levels for OS in gastrointestinal cancer

## Additional Information and Declarations

### Data Availability

The following information was supplied regarding data availability: Raw measurements are available in Supplemental Files.
