# Supplementary material for: Tissue inhibitor of metalloproteinase-1 (TIMP-1) as a prognostic biomarker in gastrointestinal cancer: a meta-analysis
Source: PeerJ. 2021 Feb 16;9:e10859. doi: 10.7717/peerj.10859 (PMC7894117; doi:10.7717/peerj.10859)
Supplement: Supplemental Information 3 [file peerj-09-10859-s003.docx]

1. The rationale for conducting the systematic review / meta-analysis.

Tissue inhibitor of metalloproteinase 1 (TIMP-1) is a major player in tumorigenesis and progression and has recently been shown to be dependent on or independent of Matrix metalloproteinases (MMPs) in its roles. This appreciation has prompted various studies assessing the prognostic value of Tissue Inhibitor of MetalloProteinase-1 (TIMP-1) in patients with gastrointestinal cancer, however, the conclusions were inconsistent. Therefore, this study aimed to assess the prognostic value of TIMP-1-immunohistochemistry (IHC) staining and pretreatment serum/plasma TIMP-1 level in gastrointestinal cancer survival as well as the association between TIMP-1 and clinicopathologic features.

2. The contribution that it makes to knowledge in light of previously published related reports, including other meta-analyses and systematic reviews.

1. Tissue inhibitors of metalloproteinases 1 (TIMP-1) has recently been shown to be dependent on or independent of MMPs in its roles in tumorigenesis and progression. Based on its complex and controversial functions, the prognostic value of TIMP-1 in gastrointestinal cancer survival was still debated. Our research found that both TIMP-1-positive IHC staining and high pretreatment serum/plasma TIMP-1 levels were significantly associated with poor survival in gastrointestinal cancer, which indicated that more possible mechanisms of TIMP-1 in gastrointestinal cancer are related to MMP-independent functions.
2. Although a recent meta-analysis of original reports demonstrated the poor prognostic value of TIMP-1-positive expression in solid cancers, the article included only 3 original studies on gastrointestinal cancer. In addition, all studies included in this meta-analysis used IHC but not ELISA to assess TIMP-1 expression in cancer patients. Moreover, it did not explore the associations between TIMP-1 and clinicopathological parameters. Therefore, we conducted a larger scale and scientifically designed meta-analysis restricted to gastrointestinal cancer to investigate the prognostic value of TIMP-1 IHC staining and pretreatment serum/plasma TIMP-1 levels and explore the associations between TIMP-1 and clinicopathological characteristics.
3. Currently, IHC and ELISA are recognized as the primary analytic methods for assessing TIMP-1 in gastrointestinal cancer. Although TIMP-1 protein levels are easily measured using ELISA, there is a substantial risk of false elevated levels due to the freezing and thawing of blood. In contrast, immunohistochemical staining on histopathologic slides is intuitive and fast but invasive. To our knowledge, no study has investigated the consistency of the prognostic value of TIMP-1 for gastrointestinal cancer survival with the use of different analytic methods. Our meta-analysis showed no significant difference in the TIMP-1 prognostic value between the two methods.
4. Based on the finding that TIMP-1 can induce chemotherapy resistance in vivo by inhibiting apoptosis, we analysed the association between serum/plasma TIMP-1 levels and OS stratified by chemotherapy status.
5. To determine the prognostic value of TIMP-1 in gastrointestinal cancer survival in subgroups restricted to country and metastasis, we performed subgroup analyses.
