# Supplementary figures and images for: Tissue inhibitor of metalloproteinase-1 (TIMP-1) as a prognostic biomarker in gastrointestinal cancer: a meta-analysis
Source: PeerJ. 2021 Feb 16;9:e10859. doi: 10.7717/peerj.10859 (PMC7894117; doi:10.7717/peerj.10859)

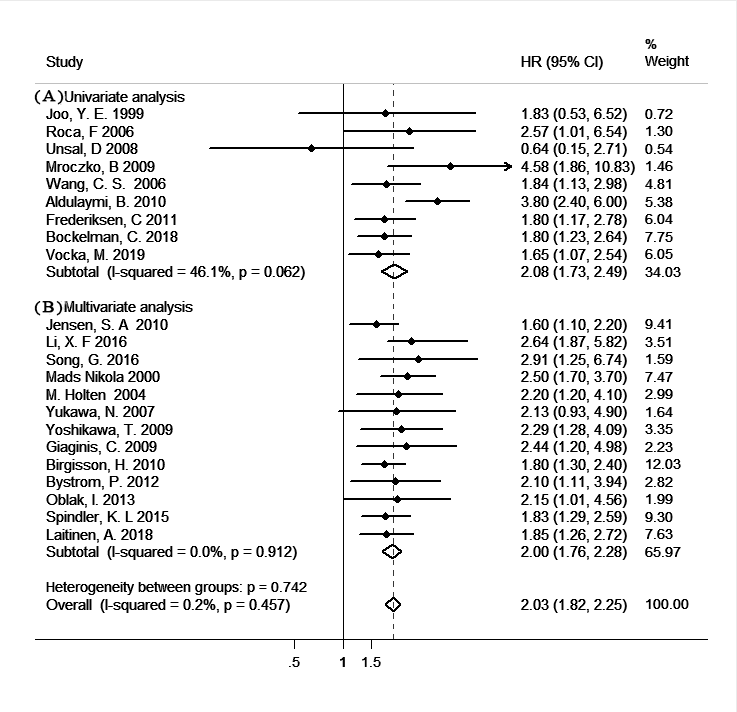

Supplement: Supplemental Information 5 — (A): univariate analysis; (B): multivariate analysis. [file peerj-09-10859-s005.png]

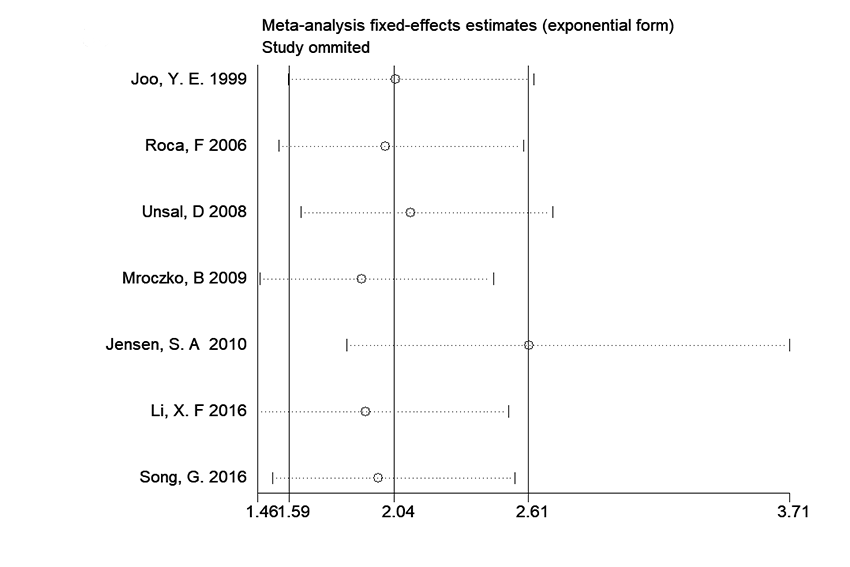

Supplement: Supplemental Information 6 [file peerj-09-10859-s006.png]

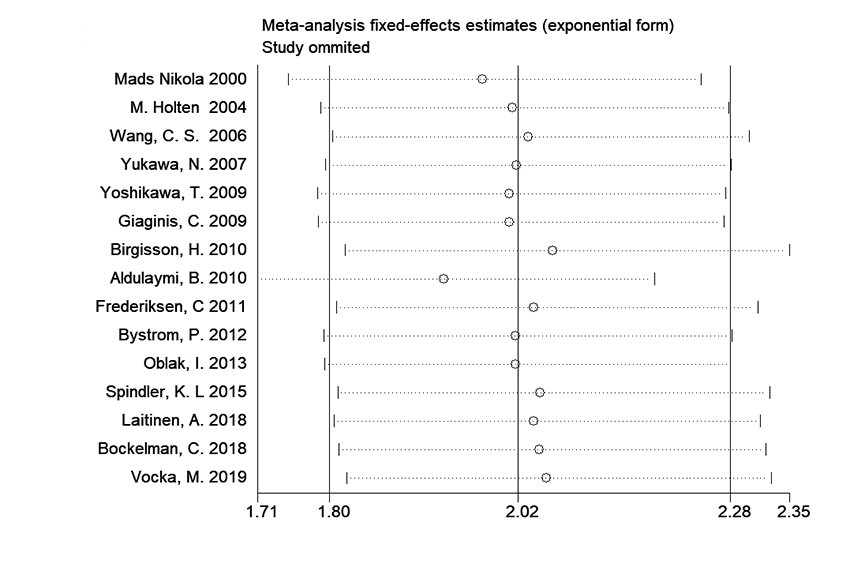

Supplement: Supplemental Information 7 [file peerj-09-10859-s007.png]

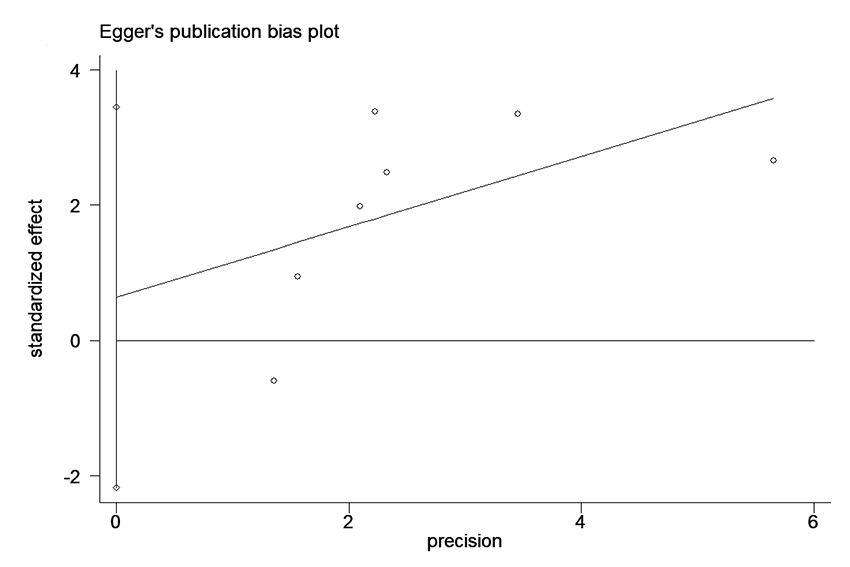

Supplement: Supplemental Information 8 [file peerj-09-10859-s008.png]

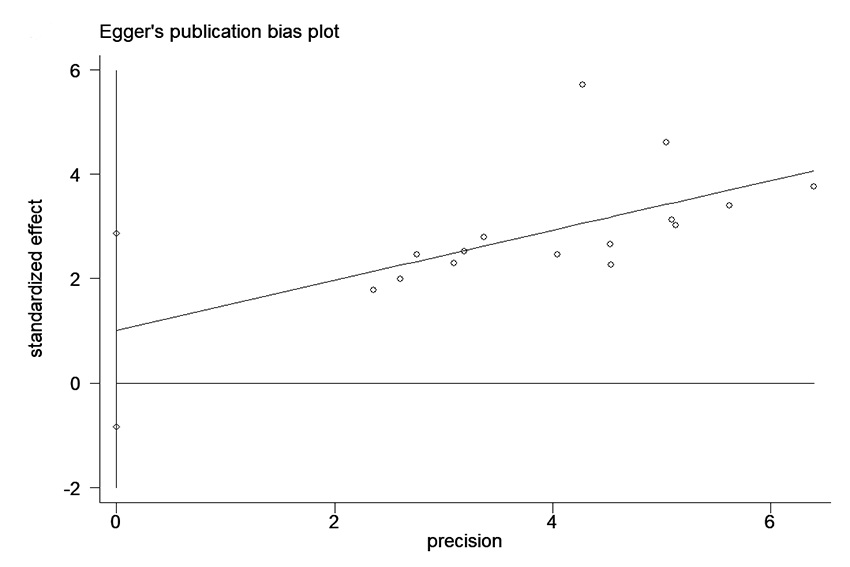

Supplement: Supplemental Information 9 [file peerj-09-10859-s009.png]
